# KRAP regulates mitochondrial Ca^2+^ uptake by licensing IP_3_ receptor activity and stabilizing ER–mitochondrial junctions

**DOI:** 10.1242/jcs.261728

**Published:** 2024-06-27

**Authors:** Peace Atakpa-Adaji, Adelina Ivanova, Karolina Kujawa, Colin W. Taylor

**Affiliations:** Department of Pharmacology, University of Cambridge, Tennis Court Road, Cambridge, CB2 1PD, UK

**Keywords:** Ca^2+^, Endoplasmic reticulum, HeLa cell, Histamine, IP_3_ receptor, KRAP, MCU, Membrane contact site, Mitochondria, Proximity ligation assay, VDAC1

## Abstract

Inositol 1,4,5-trisphosphate (IP_3_) receptors (IP_3_Rs) are high-conductance channels that allow the regulated redistribution of Ca^2+^ from the endoplasmic reticulum (ER) to the cytosol and, at specialized membrane contact sites (MCSs), to other organelles. Only a subset of IP_3_Rs release Ca^2+^ to the cytosol in response to IP_3_. These ‘licensed’ IP_3_Rs are associated with Kras-induced actin-interacting protein (KRAP, also known as ITPRID2) beneath the plasma membrane. It is unclear whether KRAP regulates IP_3_Rs at MCSs. We show, using simultaneous measurements of Ca^2+^ concentration in the cytosol and mitochondrial matrix, that KRAP also licenses IP_3_Rs to release Ca^2+^ to mitochondria. Loss of KRAP abolishes cytosolic and mitochondrial Ca^2+^ signals evoked by stimulation of IP_3_Rs via endogenous receptors. KRAP is located at ER–mitochondrial membrane contact sites (ERMCSs) populated by IP_3_R clusters. Using a proximity ligation assay between IP_3_R and voltage-dependent anion channel 1 (VDAC1), we show that loss of KRAP reduces the number of ERMCSs. We conclude that KRAP regulates Ca^2+^ transfer from IP_3_Rs to mitochondria by both licensing IP_3_R activity and stabilizing ERMCSs.

## Teaser

**Summary:** KRAP regulates transfer of Ca^2+^ from IP_3_ receptors to mitochondria by both stabilizing membrane contact sites between the ER and mitochondria and licensing IP_3_ receptors to respond to IP_3_ at these junctions.

## INTRODUCTION

Inositol 1,4,5-trisphosphate (IP_3_) receptors (IP_3_Rs) are Ca^2+^ channels expressed in the membranes of the endoplasmic reticulum (ER), where their opening, after binding of IP_3_ and Ca^2+^, allows Ca^2+^ to leak rapidly from the ER (Foskett et al., 2007; Prole and Taylor, 2019b). The Ca^2+^ released can then diffuse into the cytosol to increase the cytosolic Ca^2+^ concentration, which can then regulate diverse cellular activities. Ca^2+^ can also be delivered to other membranes to which the ER is closely apposed at membrane contact sites (MCSs) (Helle et al., 2013; Roest et al., 2017). Opening of IP_3_Rs within MCSs between the ER and lysosomes or mitochondria, for example, delivers Ca^2+^ locally at concentrations sufficient to fuel uptake by the low-affinity Ca^2+^ uptake systems of these organelles (De Stefani et al., 2011; Atakpa et al., 2018). The most thoroughly studied of these exchanges occurs within ER–mitochondrial MCSs (ERMCSs), where IP_3_Rs deliver Ca^2+^ to the mitochondrial Ca^2+^ uniporter (MCU), thereby shaping cytosolic Ca^2+^ signals and regulating mitochondrial function and cell fate (Kirichok et al., 2004; Mendes et al., 2005; De Stefani et al., 2011; Rowland and Voeltz, 2012; Bartok et al., 2019; Vecellio Reane et al., 2020). Recent observations suggest that all three IP_3_R subtypes (IP_3_R1–3) localize to ERMCSs and support Ca^2+^ delivery to mitochondria (Bartok et al., 2019). ERMCSs are held together by tethers (Vecellio Reane et al., 2020), with tripartite interactions among IP_3_Rs, the anchoring cytosolic protein GRP75 (also known as HSPA9) and the voltage-dependent anion-selective channel protein 1 (VDAC1) in the outer mitochondrial membrane (OMM), most thoroughly implicated in regulating Ca^2+^ transfer (Gincel et al., 2001; Rapizzi et al., 2002; De Stefani et al., 2012). Disruption of ERMCSs is implicated in neurodegenerative diseases (Milakovic et al., 2006; Area-Gomez et al., 2009; Kim et al., 2010; Yu et al., 2021), metabolic disorders (Tubbs and Rieusset, 2017) and cancer (Simoes et al., 2020).

Only a fraction of the IP_3_Rs expressed in a cell (∼30% in HeLa cells) release Ca^2+^ into the cytosol in response to IP_3_; these have been described as ‘licensed’ IP_3_Rs (Thillaiappan et al., 2017). Evidence that Kras-induced actin-binding protein (KRAP; also known as IP_3_ receptor-interacting domain-containing protein 2 or ITPRID2) associates with IP_3_Rs and actin (Fujimoto et al., 2011a,b) and perhaps influences Ca^2+^ release (Fujimoto et al., 2011c) prompted further analysis of its role in licensing IP_3_Rs. KRAP is ubiquitously expressed, its amino acid sequence is well-conserved across species (Inokuchi et al., 2004; Fujimoto et al., 2007; Fujimoto and Shirasawa, 2011), its interaction with IP_3_R requires a double phenylalanine motif within the N-terminal region of KRAP (Fujimoto et al., 2011b), and loss of KRAP alters the subcellular distribution of IP_3_Rs (Fujimoto et al., 2011a; Thillaiappan et al., 2021). It is now clear that licensed IP_3_Rs are tethered in small clusters by KRAP to actin near the plasma membrane and adjacent to the sites where store-operated Ca^2+^ entry occurs (Thillaiappan et al., 2021; Vorontsova et al., 2022).

Loss of KRAP abolishes the cytosolic Ca^2+^ signals evoked by IP_3_Rs, which are initiated by KRAP-licensed IP_3_Rs parked immediately beneath the plasma membrane (PM). The functions of the many IP_3_Rs that are neither immobilized nor associated with KRAP are unresolved (Thillaiappan et al., 2021). It is not known, for example, whether the IP_3_Rs that transfer Ca^2+^ at MCSs to the cytosolic surface of organelles are, similar to the IP_3_Rs that evoke cytosolic Ca^2+^ signals, licensed by KRAP. As the positioning of IP_3_Rs within specialized ERMCSs is critical for transfer of Ca^2+^ via MCU (Kirichok et al., 2004; Mendes et al., 2005; De Stefani et al., 2011; Rowland Voeltz, 2012; Bartok et al., 2019; Vecellio Reane et al., 2020), we assessed whether KRAP also regulates the IP_3_Rs that transport Ca^2+^ at these MCSs. The role of KRAP at ERMCSs has not hitherto been studied.

Here, we show that KRAP both regulates the activity of the IP_3_Rs that deliver Ca^2+^ to mitochondria and structurally stabilizes the ERMCSs. We conclude that KRAP exerts a dual regulation of Ca^2+^ transfer from ER to mitochondria.

## RESULTS

### IP_3_-evoked cytosolic and mitochondrial Ca^2+^ signals require KRAP

**Fig. 1.**
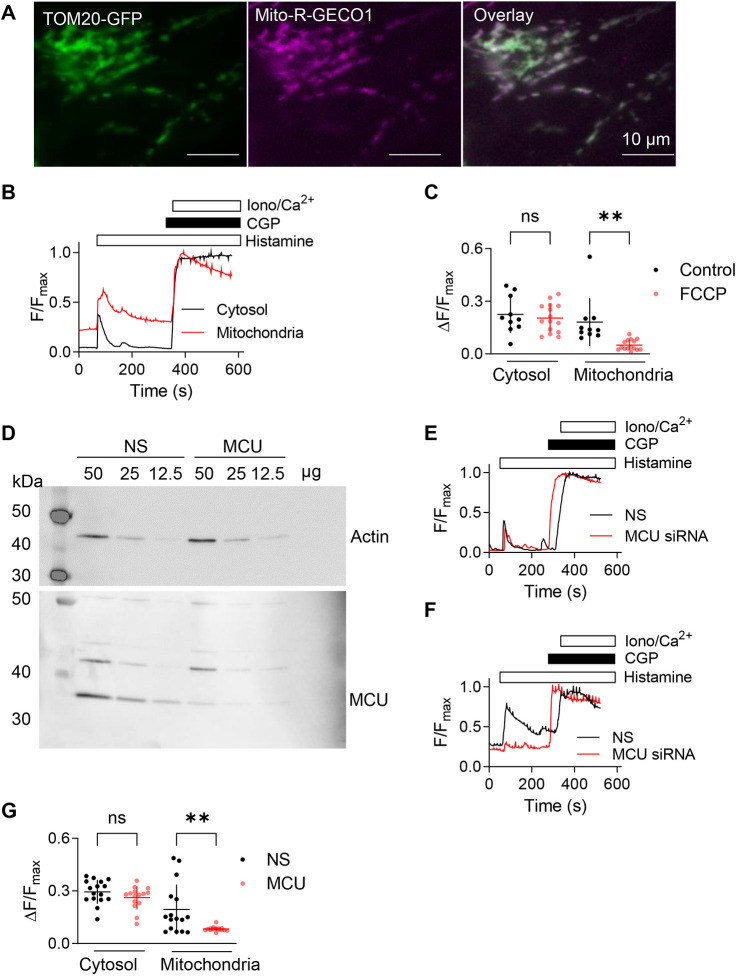
**Histamine evokes transfer of Ca^2+^ from the ER to mitochondria through IP_3_Rs.** (A) Wide-field fluorescence images of HeLa cells expressing TOM20–GFP (green) and Mito-R-GECO1 (pseudo-coloured in magenta); the overlay shows colocalization in white. Images are typical of four independent experiments. Manders' coefficients for the fraction of Mito-R-GECO1 colocalizing with TOM20–GFP=0.88±0.07 (mean±s.d.), *n*=4. (B) HeLa cells expressing Mito-R-GECO1 (mitochondrial Ca^2+^ indicator) and loaded with Calbryte 520 (cytosolic Ca^2+^ indicator) were stimulated with histamine (100 µM) in Ca^2+^-free HBS. Fluorescence from the Ca^2+^-saturated indicators (F_max_) was determined by addition of CGP 37157 (CGP, 50 µM) to inhibit the mitochondrial Na^+^/Ca^2+^ exchanger and then ionomycin (Iono, 10 μM) with 20 mM CaCl_2_. Results (F/F_max_) show fluorescence recorded from an entire cell. Results are typical of at least ten experiments. (C) Summary results show peak changes in fluorescence (ΔF/F_max_) evoked by histamine (100 µM) alone, or after pre-incubation with FCCP (5 µM, 10 min). Results show values for individual cells and mean±s.d. from ten (control) or 15 cells (FCCP) from three independent experiments. ns, not significant, *P*>0.05; **P<0.01; one-way ANOVA with Tukey's correction. (D) Western blots showing the effect of non-silencing (NS) siRNA or siRNA directed at MCU in HeLa cells. Protein loadings (μg/lane) and molecular mass markers (kDa) are shown. Results are typical of three independent experiments. MCU siRNA reduced MCU expression to 22±7% of its expression in cells treated with NS siRNA (mean±s.e.m., *n*=3). (E,F) Effects of histamine (100 µM) in Ca^2+^-free HBS on [Ca^2+^]_c_ (Calbryte 520) (E) or [Ca^2+^]_m_ (Mito-R-GECO1) (F) in single HeLa cells treated with NS or MCU siRNA. Results, reported as F/F_max_, are typical of 15–16 cells from three independent experiments. (G) Summary results show effects of histamine in HeLa cells treated with NS or MCU siRNA. Results (individual values, mean±s.d.) are from 16 (NS) or 15 cells (MCU siRNA) from three independent experiments. ns, not significant, *P*>0.05; **P<0.01; one-way ANOVA with Tukey’s correction.

We used validated Ca^2+^ indicators to simultaneously record the free Ca^2+^ concentrations in the cytosol ([Ca^2+^]_c_) and mitochondrial matrix ([Ca^2+^]_m_) of single HeLa cells (Fig. 1A; Fig. S1; Materials and Methods). Stimulation of HeLa cells in Ca^2+^-free HEPES-buffered saline (HBS) with a maximally effective concentration of histamine to evoke IP_3_ formation through endogenous receptors caused a rapid increase in both [Ca^2+^]_c_ and [Ca^2+^]_m_ (Fig. 1B). Pre-treatment of cells with carbonyl cyanide-*p*-trifluoromethoxyphenylhydrazone (FCCP) to dissipate the H^+^ gradient across the inner mitochondrial membrane had no effect on the histamine-evoked increase in [Ca^2+^]_c_, but it almost abolished the increase in [Ca^2+^]_m_ (Fig. 1C). We used siRNA to reduce expression of MCU by 78±7% (mean±s.e.m.) (Fig. 1D). This treatment almost abolished the histamine-evoked increase in [Ca^2+^]_m_ without significantly affecting the increase in [Ca^2+^]_c_ (Fig. 1E–G). These results, which are consistent with many published observations (Rapizzi et al., 2002; De Stefani et al., 2011, 2012; Bartok et al., 2019; Katona et al., 2022), confirm that IP_3_-evoked Ca^2+^ release causes rapid uptake of Ca^2+^ into mitochondria via MCU.

**Fig. 2.**
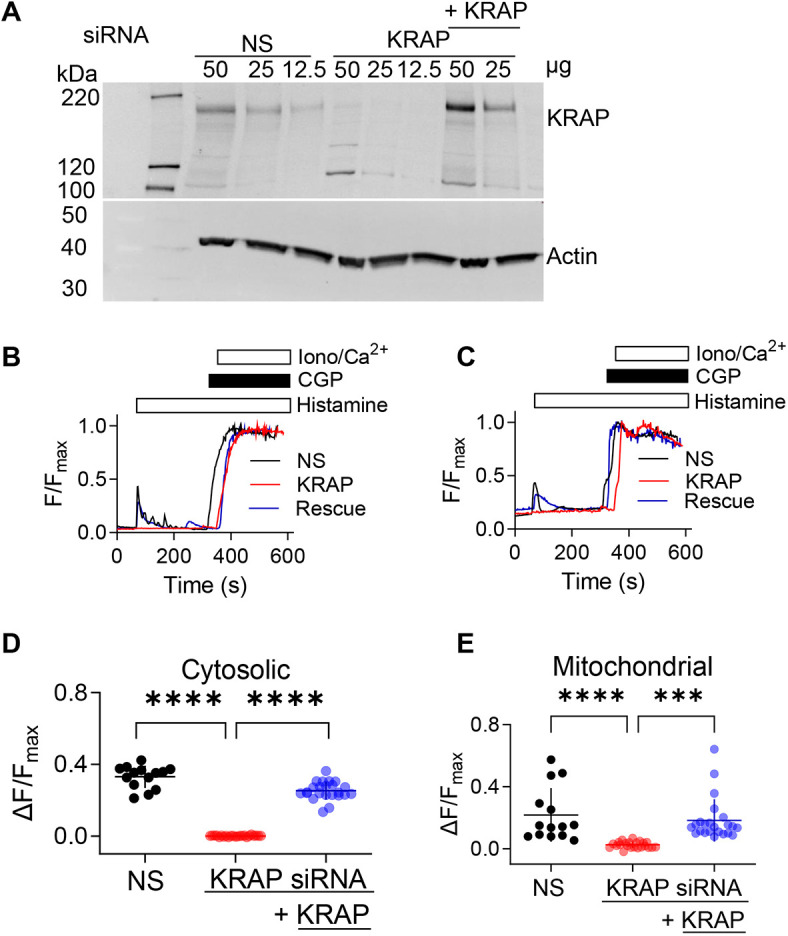
**KRAP is required for transfer of Ca^2+^ from the ER to mitochondria through IP_3_R.** (A) Western blots from HeLa cells showing the effect of NS siRNA or siRNA directed at KRAP alone or after expression of siRNA-resistant KRAP. Results are typical of four experiments. Protein loadings (μg/lane) and molecular mass markers (kDa, left) are shown. (B,C) Effects of histamine (100 µM) in Ca^2+^-free HBS on [Ca^2+^]_c_ (Calbryte 520) (B) or [Ca^2+^]_m_ (Mito-R-GECO1) (C) in single HeLa cells treated with NS siRNA, KRAP siRNA or KRAP siRNA with siRNA-resistant KRAP (‘rescue’). Results, reported as F/F_max_, are typical of 14–23 cells from three independent experiments. (D,E) Summary results show peak changes in fluorescence (ΔF/F_max_) from cytosolic (D) or mitochondrial (E) Ca^2+^ indicators. Results show individual values with mean±s.d. from 14 (NS siRNA), 23 (KRAP siRNA) and 23 cells (rescue) from three independent experiments. ***P<0.001; ****P<0.0001; one-way ANOVA with Tukey’s correction.

**Fig. 3.**
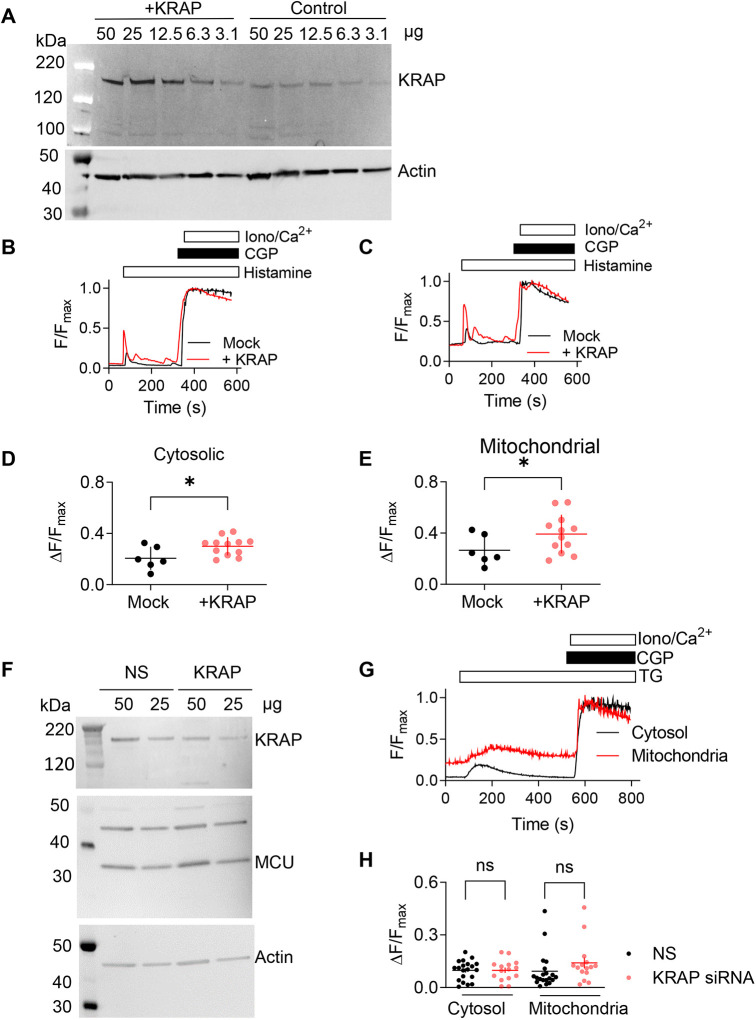
**KRAP regulates IP_3_R-mediated transfer of Ca^2+^ from the ER to mitochondria without affecting MCU.** (A) Western blots from control HeLa cells and after overexpression of KRAP. KRAP expression was 3.5±0.3-fold greater in cells overexpressing KRAP (mean±s.e.m., three independent analyses). Protein loadings (μg/lane) and molecular mass markers (kDa) are shown. (B,C) Effects of histamine (100 µM) in Ca^2+^-free HBS on [Ca^2+^]_c_ (Calbryte 520) (B) or [Ca^2+^]_m_ (Mito-R-GECO1) (C) in single HeLa cells overexpressing KRAP or after mock transfection with an empty vector. (D,E) Summary results show peak changes in cytosolic (D) and mitochondrial (E) fluorescence from Ca^2+^ indicators evoked by histamine (100 µM) in HeLa cells overexpressing KRAP or after mock transfection. Individual values with mean±s.d. are shown from six (mock) or 12 (KRAP) cells. Cells were transfected with plasmids expressing Mito-R-GECO and KRAP (in a 1:3 ratio), and all cells expressing Mito-R-GECO in the field were included in the analysis. *P<0.05; two-tailed unpaired Student's *t*-test. (F) Western blots from HeLa cells showing the effect of NS siRNA or siRNA directed against KRAP on the expression of MCU. KRAP expression was reduced by 70±6% (mean±s.e.m.). MCU expression was 97±24% of its level in NS siRNA-treated cells. *P*>0.05; two-tailed paired Student's *t*-test of MCU expression in KRAP siRNA-treated cells compared to that in NS siRNA-treated cells from six independent experiments. (G) HeLa cells expressing Mito-R-GECO1 and loaded with Calbryte 520 were stimulated with thapsigargin (TG, 5 µM) in Ca^2+^-free HBS, and then CGP 37157 (CGP, 50 µM) and ionomycin (Iono, 10 μM) with 20 mM CaCl_2_ to obtain F_max_. Results (F/F_max_) show fluorescence recorded from an entire cell for each indicator. Results are typical of at least three experiments. (H) Summary results show peak changes in fluorescence (ΔF/F_max_) from cytosolic or mitochondrial Ca^2+^ indicators evoked by thapsigargin (5 µM). Mean±s.d. (and individual values) are shown from 20 (NS siRNA) and 15 (KRAP siRNA) cells from three independent experiments. ns, not significant, *P*>0.05; one-way ANOVA with Tukey’s correction.

Treatment of HeLa cells with an appropriate siRNA caused a substantial (83±9%, mean±s.e.m.) reduction in KRAP expression (Fig. 2A) and abolished the histamine-evoked increases in both [Ca^2+^]_c_ and [Ca^2+^]_m_. Both responses were rescued by expression of a siRNA-resistant KRAP (Fig. 2B–E). Furthermore, overexpression of KRAP in HeLa cells (352±32% of control levels, mean±s.e.m.) exaggerated the histamine-evoked increases in both [Ca^2+^]_c_ and [Ca^2+^]_m_ (Fig. 3A–E). We conclude that both the global cytosolic Ca^2+^ signals evoked by IP_3_ and the uptake of Ca^2+^ by mitochondria through MCU require KRAP.

### KRAP is required for delivery of Ca^2+^ from IP_3_R to mitochondria

We next considered whether the requirement for KRAP for mitochondrial Ca^2+^ uptake arises from KRAP regulating delivery of Ca^2+^ to the OMM and/or regulation of MCU. Treatment of HeLa cells with KRAP siRNA had no effect on MCU expression [MCU expression was 97±24% of its level in non-silencing (NS) siRNA-treated cells, mean±s.e.m., *n*=6] (Fig. 3F) or mitochondrial membrane potential (Fig. S2). HeLa cells were stimulated with thapsigargin in Ca^2+^-free HBS to promote loss of Ca^2+^ from the ER, which is independent of IP_3_R activity. Thapsigargin evoked increases in both [Ca^2+^]_c_ and [Ca^2+^]_m_, but neither response was affected by siRNA-mediated loss of KRAP expression (Fig. 3G,H**)**.

These results establish that KRAP is required for mitochondria to accumulate Ca^2+^ released through IP_3_Rs. Furthermore, the deficient Ca^2+^ transfer in the absence of KRAP is not due to collapse of the mitochondrial membrane potential, loss of MCU expression or compromised function of MCU.

### KRAP mediates association of IP_3_R with mitochondria

**Fig. 4.**
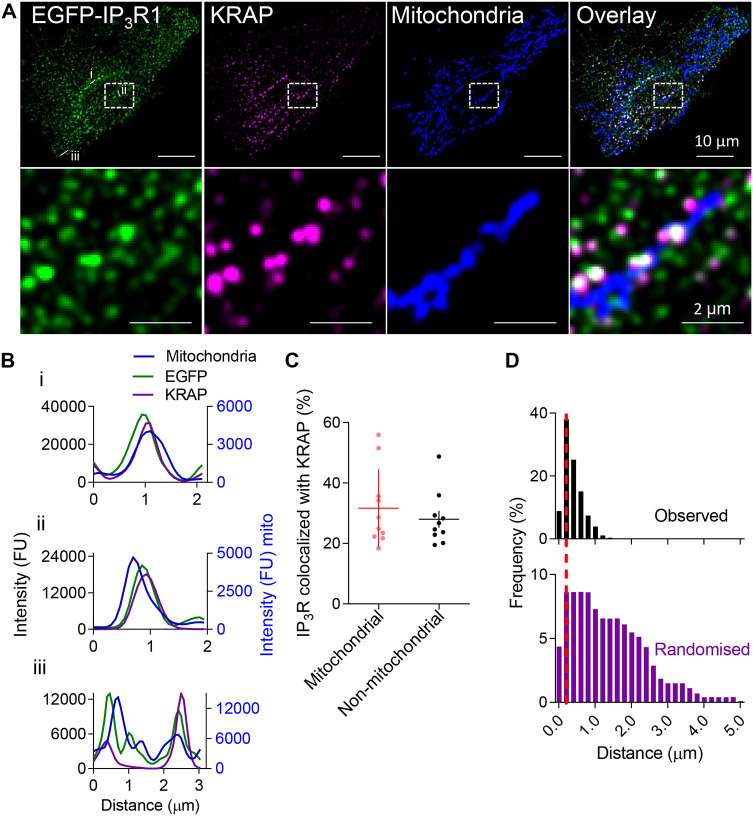
**Colocalization of KRAP and IP_3_R with mitochondria.** (A) Confocal section near the plasma membrane of an EGFP–IP_3_R1 HeLa cell loaded with MitoTracker Deep Red (pseudo-coloured in blue) and immunostained for KRAP (magenta). GFP Booster was used to enhance the fluorescence intensity of the EGFP signal. Images show some IP_3_R puncta (green) colocalized with KRAP (white where colocalized) associated with mitochondria. Boxed regions are enlarged below. Images are typical of ten cells. (B) Fluorescence intensity profiles of EGFP–IP_3_R1, KRAP and mitochondria from the regions marked by white lines and labelled i–iii in A. FU, fluorescence units. (C) Percentage of mitochondria-associated IP_3_R puncta or non-mitochondria-associated IP_3_R puncta that colocalize with KRAP (centre-to-centre separations <233 nm). Results show individual values with mean±s.d. for ten cells. *P*>0.05; two-tailed paired Student's *t*-test. (D) Frequency distributions of centre-to-centre distances for mitochondria-associated IP_3_R puncta (2071 puncta from ten cells) and the nearest KRAP punctum. Observed values and values after randomization of positions of the KRAP puncta (100 iterations) are shown. 31.7±12.9% (mean±s.d., *n*=10 cells) of IP_3_R puncta are within 233 nm (red line) of a KRAP punctum (5.2±2.7% after randomization; *P*<0.0001, two-tailed paired Student's *t*-test). Images and results are representative of ten independent dishes.

We used HeLa cells in which endogenous IP_3_R1 (encoded by *ITPR1*) was tagged with EGFP (EGFP–IP_3_R1 HeLa cells), an antibody to KRAP and MitoTracker to explore the relationships among IP_3_Rs, mitochondria and KRAP. Although only IP_3_R1 is visible in EGFP–IP_3_R1 HeLa cells, it is the major IP_3_R subtype in these cells (Itzhak et al., 2016) and it assembles into tetrameric channels with the other subtypes (Thillaiappan et al., 2017). We assume, therefore, that EGFP identifies most IP_3_Rs in EGFP–IP_3_R1 HeLa cells (Thillaiappan et al., 2021). Confocal microscopy established that 24±11% of EGFP–IP_3_Rs (mean±s.d., *n*=10 cells) were associated with mitochondria and many of these IP_3_R puncta were associated with KRAP (Fig. 4A,B). Quantitative analyses using an object-based method (Gilles et al., 2017) established that the colocalization of KRAP with IP_3_Rs was indistinguishable for IP_3_Rs associated with mitochondria (32±13%, mean±s.d., *n*=10) and for IP_3_Rs with no mitochondrial association (28±9%, *n*=10) (Fig. 4C). Analysis of nearest-neighbour distances revealed that IP_3_Rs affiliated with mitochondria were significantly more colocalized with KRAP compared to randomly distributed IP_3_R puncta (Fig. 4D). These results establish that a significant fraction of the IP_3_Rs that associate with mitochondria are also associated with KRAP. Our conclusion is consistent with a proteomic analysis that identified KRAP near the OMM (Hung et al., 2017).

**Fig. 5.**
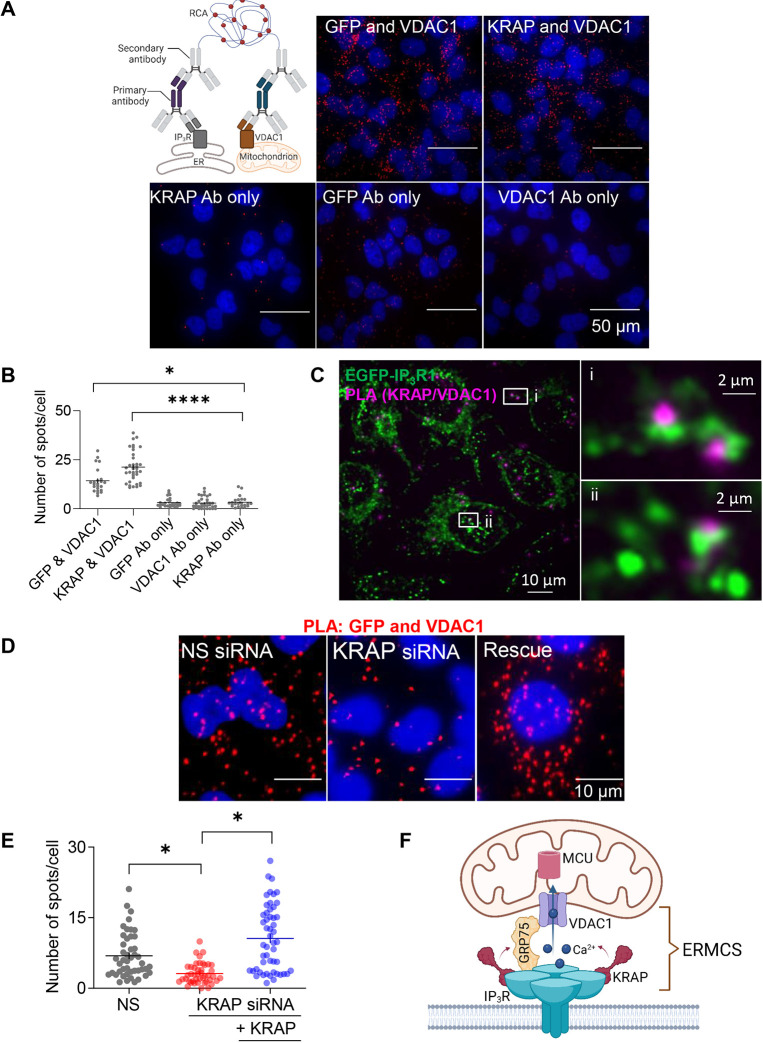
**KRAP contributes to the association of IP_3_R with mitochondria.** (A) Proximity ligation assay (PLA) uses primary antibodies that recognize proteins in the ER or mitochondrial membranes. Hybridization of complementary oligonucleotides conjugated to secondary antibodies occurs if the primary antibodies are within ∼40 nm of each other. Ligation of the hybridized strands then allows rolling circle amplification (RCA) of the oligonucleotide and incorporation of the red fluorescent probe. PLA analyses of EGFP–IP_3_R1 HeLa cells used combinations of antibodies (Ab) to VDAC1, KRAP or GFP (for EGFP–IP_3_R1). Maximum-intensity *z*-projections of confocal images show the PLA dots (red) and nuclei (DAPI, blue). Control images with only single antibodies are also shown. Results are typical of three to five independent PLA analyses. (B) Summary results show the average number of PLA dots per cell in a field that typically included ∼10 cells. Results show values for each field with mean±s.e.m. For each analysis, the number of cells, fields and independent experiments were: GFP and VDAC1 (512, 23, 4), KRAP and VDAC1 (983, 37, 6), KRAP only (559, 23, 3), GFP only (767, 26, 3) and VDAC1 only (738, 34, 4). *P<0.05; ****P<0.0001; one-way ANOVA with Tukey’s correction. (C) Confocal section of PLA in EGFP–IP_3_R1 HeLa cells shows KRAP proximity to VDAC1 (PLA dots, magenta) and endogenously tagged IP_3_R1 (green). Enlargements of boxed areas show coincidence of EGFP–IP_3_R puncta with PLA spots. Manders' split coefficient, 0.82±0.15 (mean±s.d.); *n*=7 cells. (D) PLA analyses of the interactions between VDAC1 and EGFP–IP_3_R1 in EGFP–IP_3_R1 HeLa cells treated with NS siRNA or KRAP siRNA and after rescue with an siRNA-resistant KRAP expression plasmid. Maximum-intensity *z*-projections of confocal images show the PLA dots (red) and nuclei (DAPI, blue). (E) Summary results show the average number of PLA dots per cell in a field, with mean±s.e.m. For each treatment, the number of cells, fields and independent experiments were: NS siRNA (1116, 46, 7), KRAP siRNA (1003, 44, 7) and rescue (753, 51, 7). *P<0.05; repeated measures one-way ANOVA with Bonferroni's test performed on the seven matched experiments. (F) IP_3_Rs at ER–mitochondrial membrane contact sites (ERMCSs) release Ca^2+^ that fuels the low-affinity mitochondrial Ca^2+^ uptake system (MCU). KRAP licenses these IP_3_Rs to release Ca^2+^ and it also stabilizes the ERMCS.

We used proximity ligation assays (PLAs), which detect proteins closer than about 40 nm (Tubbs and Rieusset, 2016), to examine the association of VDAC1, a protein embedded in the OMM, with IP_3_Rs and KRAP. The results demonstrate that VDAC1 is in close proximity to both IP_3_Rs and KRAP in EGFP–IP_3_R1 HeLa cells (Fig. 5A,B). Furthermore, EGFP–IP_3_R puncta and the PLA spots indicative of KRAP and VDAC1 proximity were significantly colocalized (Manders' split coefficient, 0.82±0.15; *n*=7 cells, Costes' *P*-value, 100%) (Fig. 5C). These results confirm that many IP_3_Rs that are affiliated with mitochondria are closely associated with KRAP.

Next, we used PLA with EGFP–IP_3_R1 and VDAC1 to determine the effects of KRAP on the association of IP_3_Rs with mitochondria. siRNA-mediated knockdown of KRAP caused a significant reduction in the PLA signal. The association of IP_3_Rs with mitochondria was restored by the expression of siRNA-resistant KRAP (Fig. 5D,E). These results establish that KRAP contributes to maintaining the ERMCSs wherein IP_3_Rs and mitochondria interact to facilitate the delivery of Ca^2+^ from the ER to mitochondria (Fig. 5F).

## DISCUSSION

We showed previously that IP_3_Rs must be licensed by their association with KRAP before they can respond to IP_3_ and evoke local or global cytosolic Ca^2+^ signals (Thillaiappan et al., 2021). Our present results extend these observations by demonstrating that KRAP is also required to license the IP_3_Rs that deliver Ca^2+^ to the surface of mitochondria within ERMCSs (Figs 1–3). KRAP is present at ERMCSs, a substantial fraction of mitochondria-associated IP_3_Rs (∼30%) colocalize with KRAP, and loss of KRAP abolishes the mitochondrial Ca^2+^ uptake that usually follows activation of IP_3_Rs (Figs 1–5). As the loss of mitochondrial Ca^2+^ uptake is not due to collapse of the mitochondrial membrane potential (Fig. S2) or deficient MCU (Fig. 3G,H), we suggest that regardless of whether IP_3_Rs deliver Ca^2+^ to the cytosol or within ERMCSs, their activity must be licensed by association with KRAP (Fig. 5F). Within both the cytosol and ERMCSs, only ∼30% of IP_3_Rs are associated with KRAP, suggesting that, in both locations, most IP_3_Rs are incapable of mediating Ca^2+^ release.

We note in passing that dissipating the mitochondrial membrane potential (with FCCP) or inhibition of MCU expression abolished IP_3_-evoked mitochondrial Ca^2+^ uptake, but there was no associated ‘overflow’ increase in [Ca^2+^]_c_ (Fig. 1C–G). Similar results have been reported by others (Young et al., 2022), although, more commonly, inhibition of mitochondrial Ca^2+^ uptake has been reported to exaggerate the IP_3_-evoked increase in [Ca^2+^]_c_ (Drago et al., 2012; Fonteriz et al., 2016; Koval et al., 2019; Lombardi et al., 2019). The disparity probably reflects the differential distribution of licensed IP_3_Rs between ERMCSs and cytosol-disposed ER in different cells; only cells with many of their licensed IP_3_Rs within ERMCSs are likely to show detectable ‘overflow’ increases in [Ca^2+^]_c_ when mitochondrial Ca^2+^ uptake is inhibited.

Our results identified an additional role for KRAP in facilitating Ca^2+^ transfer at ERMCSs, namely in maintaining the assembly of the ERMCSs wherein Ca^2+^ transfer from IP_3_Rs to mitochondria occurs. PLA analyses of KRAP with VDAC1 (in the OMM) showed that they were closely associated (within ∼40 nm of each other) at ERMCSs, and similar analyses with VDAC1 and IP_3_Rs established that their association was disrupted when KRAP expression was reduced (Fig. 5). The PLA analyses were restricted to endogenously tagged EGFP–IP_3_R1 because the GFP antibody is better than any available IP_3_R antibodies. However, our results are likely to be applicable to all three IP_3_R subtypes. First, within the HeLa cells used for PLA, the association of EGFP–IP_3_R1 with the other subtypes is consistent with their random assembly into tetramers (Thillaiappan et al., 2017). Second, recent work shows that all three IP_3_R subtypes contribute to Ca^2+^ transfer between the ER and mitochondria (Bartok et al., 2019; Katona et al., 2022). We have not therefore assessed whether KRAP differentially regulates IP_3_R subtypes at ERMCSs.

We suggest that KRAP is required for effective assembly or for maintaining the stability of ERMCSs wherein IP_3_R delivers Ca^2+^ to mitochondria (Fig. 5F). This observation aligns with a developing theme in Ca^2+^ signalling, namely that the Ca^2+^ channels responsible for delivering Ca^2+^ into MCSs might independently contribute to the structural stability of the MCSs. IP_3_Rs, for example, have been reported to contribute to the stability of ERMCSs (Bartok et al., 2019), and the endosomal Ca^2+^ channel TPC1 (also known as TPCN1) has been reported to stabilize the MCS between the ER and endosomes (Kilpatrick et al., 2017). Within ERMCSs, the structural contribution of IP_3_Rs to MCS stability occurred even with pore-dead Ca^2+^ channels (Bartok et al., 2019; Katona et al., 2022). Hence, the ∼70% of IP_3_Rs at ERMCSs that are not associated with KRAP and thus incapable of releasing Ca^2+^ might nevertheless contribute to assembling the ERMCSs. Our results develop this theme further by revealing that KRAP, a protein essential for IP_3_R activity, also contributes to the structural stability of ERMCSs. IP_3_Rs can move in and out of ERMCSs, but they must be immobilized to effectively transfer Ca^2+^ to mitochondria (Katona et al., 2022). Our results suggest that KRAP, in addition to its role in licensing IP_3_Rs beneath the PM (Thillaiappan et al., 2017, 2021), also immobilizes and licenses IP_3_Rs deeper within the cell at ERMCSs. We have not resolved whether the enhanced stability of ERMCSs by KRAP is mediated by immobilized IP_3_Rs or by other interactions between KRAP and the ER and/or mitochondrial proteins.

A protein related to KRAP, TESPA1 (thymocyte-expressed, positive selection-associated 1), which shares an IP_3_R-binding motif with KRAP, also interacts with IP_3_Rs at ERMCSs and regulates cytosolic and mitochondrial Ca^2+^ signals (Matsuzaki et al., 2013). However, unlike the ubiquitously expressed KRAP (Fujimoto et al., 2007), TESPA1 is expressed predominantly in immune cells (Matsuzaki et al., 2012), such that a widespread regulation of ERMCSs by TESPA1 is unlikely.

We conclude that KRAP, originally identified because it is upregulated in colorectal cancer cell lines expressing mutant KRas (Inokuchi et al., 2004), fulfils a dual function in regulating Ca^2+^ transfer from the ER to mitochondria through IP_3_Rs. KRAP licenses IP_3_R within ERMCSs to respond to IP_3_, just as it does for IP_3_Rs that release Ca^2+^ to the cytosol (Thillaiappan et al., 2021). In addition, KRAP contributes to the structural stability of the ERMCSs wherein the Ca^2+^ transfer from the ER to mitochondria occurs. Remodelling of mitochondrial Ca^2+^ signalling is critical in shaping Ras-dependent oncogenic signalling cascades (Rimessi et al., 2015). Therefore, our findings also link the regulation of IP_3_R-mediated Ca^2+^ delivery at ERMCSs to Ras signalling.

## MATERIALS AND METHODS

### Materials

Calbryte 520 AM was from AAT Bioquest (Sunnyvale, CA, USA). Histamine dihydrochloride, Triton X-100, FCCP, ECL western blotting detection reagents (Cytiva) and poly-L-lysine (0.1% w/v in H_2_O) were from Sigma-Aldrich. Bovine serum albumin (BSA) was from Europa Bio-Products (Ely, UK). Human fibronectin was from Merck Millipore (Watford, UK). CGP 37157 [7-chloro-5-(2-chlorophenyl)-1,5-dihydro-4,1-benzothiazepin-2(3H)-one] and ionomycin were from Cambridge Biosciences (Cambridge, UK). MitoTracker Deep Red FM, NuPAGE 4–12% Bis–Tris gels (NB0322BOX), iBlot 2 transfer stacks, polyvinyl difluoride (PVDF) regular size membranes (IB24001), iBlot gel transfer system, PBS and the Neon transfection system were from Thermo Fisher Scientific. TransIT-LT1 reagent was from Geneflow (Lichfield, UK). Imaging dishes were from Cellvis (Gerasdorf bei Wein, Austria). Plasmids encoding the following proteins were used: TOM20 [translocase of outer (mitochondrial) membrane 20, also known as TOMM20] tagged to GFP (TOM20–GFP) (Prole and Taylor, 2019a), Mito-R-GECO1 (Addgene, #46021) (Wu et al., 2013), human KRAP with an N-terminal myc-DDK tag (OriGene, Rockville, MD, USA, #RC205550) and siRNA-resistant human KRAP with an N-terminal myc-DDK tag (Thillaiappan et al., 2021). The following antibodies were used for western blotting (WB), immunocytochemistry (IC), or PLAs: anti-KRAP (rabbit polyclonal; ProteinTech, Manchester, UK, #14157-1-AP, RRID:AB_2195472; WB 1:1000), ChromoTek GFP-Booster ATTO488 (nanobody; Chromotek, Planegg-Martinsried, Germany #gba488; RRID: AB_2631386; IC 1:500), anti-MCU (rabbit polyclonal; Thermo Fisher Scientific, PA5-109304, RRID:AB_2854715; WB 1:1000), anti-β-actin (mouse monoclonal; Cell Signaling Technology, Leiden, Netherlands, #8H10D10, RRID:AB_2242334; WB 1:20,000), IRDye 680RD goat anti-mouse IgG secondary antibody (LI-COR, 926-68070, RRID:AB_10956588; WB 1:10,000), horseradish peroxidase (HRP)-conjugated sheep anti-rabbit IgG H&L (Abcam, ab6795, RRID:AB_955446; WB 1:10,000), Alexa Fluor 568-conjugated goat anti-rabbit IgG (H+L) (Thermo Fisher Scientific, A-11011, RRID:AB_143157; WB 1:10,000), Alexa Fluor 488-conjugated goat anti-rabbit IgG (H+L) (Thermo Fisher Scientific, A-11008, RRID:AB_143165; WB 1:10,000), Alexa Fluor 568-conjugated goat anti-mouse IgG (H+L) (Thermo Fisher Scientific, A-21235, RRID:AB_2535804; WB 1:10,000), anti-VDAC1 (Abcam, ab14734, RRID:AB_443084; PLA 1:400), anti-GFP (Abcam, ab290, RRID:AB_303395; PLA 1:200, WB 1:1000) and anti-TOM20 (Abcam, ab56783, RRID:AB_945896; WB 1:1000). The PLA kits used were Duolink *in situ* PLA probes (anti-rabbit PLUS and anti-mouse MINUS, affinity-purified donkey anti-rabbit and anti-mouse IgG, respectively), Duolink *in situ* detection reagents Red, mounting medium containing DAPI and wash buffer, from Sigma-Aldrich or NaveniFlex 100 MR (from Cambridge Biosciences). The siRNAs used were: Silencer siRNA against human KRAP (Thermo Fisher Scientific, #AM16708, assay ID, 143004), Silencer negative control no.1 (Thermo Fisher Scientific, #AM4635) and ON-TARGETplusTM SMARTpool MCU siRNA (Dharmacon, L-015519-02-0005). All siRNA sequences are provided in Table S1. The Bradford protein assay kit (DC protein assay kit II, 5000112) and MOPS SDS Running Buffer 20× (NP001-02) were from Bio-Rad. Tetramethylrhodamine ethyl ester (TMRE)-mitochondrial membrane potential assay kit was from Abcam (ab113852). The sources of additional materials are provided in relevant sections of the Materials and Methods.

### Cell culture and transfection

HeLa and edited HeLa cells, in which both copies of the endogenous genes encoding IP_3_R1 (*ITPR1*) were N-terminally tagged with monomeric EGFP (EGFP–IP_3_R1 HeLa cells) (Thillaiappan et al., 2017), were cultured in Dulbecco's modified Eagle medium (DMEM)/F-12 with GlutaMAX (Thermo Fisher Scientific, #31331028) and 10% foetal bovine serum (FBS; Sigma-Aldrich, #F7524, batch BCBX5042). The cells were maintained at 37°C in humidified air with 5% CO_2_ and passaged every 3–4 days with TrypLE Express (Thermo Fisher Scientific, #12605010). Regular screening confirmed that all cells were mycoplasma free.

For imaging, cells were grown on 35-mm glass-bottomed dishes coated with human fibronectin (10 μg/ml). For transient transfections with protein-encoding plasmids, cells were transfected using TransIT-LT1 reagent (1.5 μg DNA per 3.75 μl reagent per dish) according to the manufacturer's instructions. Cells were assayed after 24 h. Transfections with siRNA followed the manufacturer's instructions using a Neon transfection system (Thermo Fisher Scientific). Briefly, ∼100,000 cells were diluted in 100 μl of R buffer and incubated with siRNA (200 nM). Cells were electroporated using a 100-μl Neon pipette tip (pulse voltage 1005 V; pulse width, 35 ms; two pulses) and transferred into complete growth medium. Electroporated cells were distributed into fibronectin-coated 35-mm imaging dishes (∼50,000 cells/dish) for microscopy or into 25-cm^2^ culture flasks (∼100,000 cells) for protein analyses. Cells were assayed after 72 h. For rescue experiments, cells were first electroporated with siRNA (200 nM), and after 48 h, they were transfected with an siRNA-resistant KRAP plasmid (4.5 μg) using TransIT-LT1 reagent. Cells were used after a further 24 h.

### Western blotting

Cells in 25-cm^2^ culture flasks were harvested by centrifugation (650 ***g***, 2 min, 22°C), washed with ice-cold PBS (1.06 mM KH_2_PO_4_, 155 mM NaCl, 3 mM Na_2_HPO_4_, pH 7.3, 20°C), and the pellet was resuspended (0.1 ml) in radioimmunoprecipitation assay buffer (RIPA; 150 mM NaCl, 1% Triton X-100, 0.5% sodium deoxycholate, 0.1% SDS, 50 mM Tris, pH 8.0) containing protease inhibitors (Sigma-Aldrich, #11873580001; 1 mini-tablet per 10 ml). After sonication on ice (Transsonic, T420; three 10 s sonication pulses and three 10 s pauses), samples were incubated with rotation (30 min, 4°C) and centrifuged (14,000 ***g***, 15 min, 4°C), and the supernatant was used for analysis. Proteins were quantified using the DC protein assay kit II with BSA in RIPA buffer as the standard. For western blotting, proteins were separated on NuPAGE 4–12% Bis-Tris gels, run at 160 V for 45 min in MOPS SDS running buffer, and transferred to a PVDF membrane using an iBlot 2 gel-transfer system. The membrane was blocked (1 h with shaking) in TBS (137 mM NaCl, 20 mM Tris, pH 7.6) containing 0.1% Tween 20 (TBST) with 5% BSA, washed twice with TBST, incubated in TBST containing 1% BSA (16 h, 4°C with shaking) with the primary antibody, washed with TBST (five times for 5 min), incubated with secondary antibody in TBST containing 1% BSA (1 h, 20°C with shaking), and washed with TBST (five times for 5 min). Bands were quantified using an iBright FL1500 imaging system (Thermo Fisher Scientific) either directly for the fluorescent secondary antibody (Alexa Fluor 568) or by chemiluminescence after treatment with ECL Prime western blotting detection reagent (for HRP-conjugated secondary antibodies; see Materials). We confirmed that, within the range of loadings used for analysis, protein band intensities scaled linearly with the amount of protein loaded.

### Immunocytochemistry

Cells grown on fibronectin-coated 35-mm glass-bottomed dishes were washed three times with HBS (20°C) and then incubated with MitoTracker Deep Red (500 nM, 30 min, 37°C). HBS had the following composition: 135 mM NaCl, 5.9 mM KCl, 1.2 mM MgCl_2_, 1.5 mM CaCl_2_, 11.5 mM d-glucose, 11.6 mM HEPES, pH 7.3. Cells were washed three times with HBS, fixed with methanol (20 min, −20°C), washed three times with PBS, and permeabilized using 0.25% Triton X-100 in PBS (5 min, 20°C). After three washes with PBS, cells were incubated in PBS containing 5% BSA (1 h, 20°C), washed in the same medium, then incubated (12 h, 4°C) with ChromoTek GFP-Booster ATTO488 and the primary antibody to KRAP in PBS containing 3% BSA. Cells were then washed three times with PBS, incubated in the dark with the secondary antibody in PBS with 3% BSA (1 h, 20°C), and washed three times with PBS before imaging. Dilutions of the antibodies used are provided in the Materials section.

### Proximity ligation assays

A Duolink PLA was used to determine protein interactions at ERMCSs. PLA reports the presence of juxtaposed proteins when secondary antibodies conjugated to complementary oligonucleotides come sufficiently close (∼40 nm) to hybridize and allow rolling-circle amplification, which is then detected with a fluorescent probe (Texas Red) (Tubbs and Rieusset, 2016) (Fig. 5A). Cells grown on fibronectin-coated 35-mm glass-bottomed dishes were fixed in PBS with 4% paraformaldehyde (30 min, 20°C), washed with PBS, permeabilized (0.25% Triton X-100 in PBS, 5 min, 20°C) and incubated with primary antibodies (16 h, 4°C). All subsequent steps, including incubation with the oligonucleotide-conjugated secondary antibodies (anti-rabbit PLUS and anti-mouse MINUS), were performed according to the manufacturer's instructions. The antibodies and their dilutions are described in the Materials section. PLA products were visualized using an Olympus microscope equipped with a 60× objective, and the number of spots per cell were quantified using ImageJ. PLA analysis of interactions between EGFP–IP_3_R and VDAC1 or KRAP and VDAC1 used Duolink PLA reagents (Fig. 5A,B). Supply difficulties necessitated the use of a different PLA kit (NaveniFlex) for analyses of KRAP knockdown (Fig. 5D,E). Indistinguishable results were obtained when the two kits were applied to analyses of the interactions between IP_3_R on the ER and VDAC1 on the mitochondria.

### Fluorescence microscopy

Fluorescence microscopy used an Olympus (Ca^2+^ imaging, colocalization of Mito-R-GECO and PLA analysis) or Zeiss (three-colour imaging of IP_3_R, KRAP and mitochondria) microscope. The inverted Olympus IX83 microscope was equipped with 60× (numerical aperture or NA, 1.45) and 100× oil-immersion objectives (NA, 1.49), a multi-line laser bank (488 and 561 nm) and an iLas^2^ targeted laser illumination system (Cairn, Faversham, UK). The excitation light passed through a quad dichroic beam-splitter (TRF89902-QUAD). The emitted light passed through appropriate filters (Cairn Optospin; peak/bandwidth (nm): 525/50 and 630/75) and was detected with an iXon Ultra 897 electron multiplied charge-coupled device (EMCCD) camera (512×512 pixels, Andor, Belfast, Northern Ireland). The Zeiss LSM700 upright confocal microscope, on which imaging dishes were viewed in an inverted position, was equipped with a 63× oil-immersion objective (NA, 1.4) and four excitation solid-state laser lines (405, 488, 555 and 639 nm). The emitted light was detected by two photomultiplier tubes in the scanhead using a shared pinhole and a sliding dichroic filter to separate the emitted wavelengths. We confirmed that for all multi-colour imaging, there was no bleed-through between channels.

### Measurement of cytosolic and mitochondrial Ca^2+^ concentrations in single cells

We sought cytosolic and mitochondrial Ca^2+^ indicators with compatible spectra and with changes in fluorescence amplitude after stimulation with a maximally effective concentration of histamine (100 μM) that were usually less than 50% of the maximal fluorescence signal (F_max_). F_max_ signals were determined after addition of CGP 37157 (50 µM) to inhibit the mitochondrial Na^+^/Ca^2+^ exchanger (Suzuki et al., 2014) and then, after 1 min, addition of ionomycin (10 μM) in HBS containing a final Ca^2+^ concentration of 20 mM. With these conditions satisfied (i.e. the fluorescence signal F usually less than 50% of F_max_), it is reasonable to assume that changes in the fluorescence intensity of the Ca^2+^ indicators are approximately linearly related to changes in [Ca^2+^]. After investigating several cytosolic (Fluo 8 AM, Cal 520 AM and Calbryte 520 AM) and mitochondrial Ca^2+^ indicators (Fura 2 AM, MtCEPIA 2, Mito-LAR-GECO1.2 and Mito-R-GECO1), we selected Calbryte 520 (equilibrium dissociation constant for Ca^2+^, 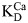
=1200 nM) and Mito-R-GECO1 (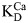
= 480 nM) (Wu et al., 2013) to measure [Ca^2+^]_c_ and [Ca^2+^]_m_, respectively.

HeLa cells grown on fibronectin-coated 35-mm glass-bottomed dishes were transfected with Mito-R-GECO1 and then loaded with Calbryte 520 by incubation with Calbryte 520 AM (2 μM, 1 h, 20°C) in HBS supplemented with 0.02% Pluronic F-127 (Sigma-Aldrich, #P2443). Cells were then washed and incubated in HBS for 30 min, and, immediately before imaging, the HBS was replaced with Ca^2+^-free HBS (HBS with no added CaCl_2_). Imaging at 20°C used an Olympus IX83 microscope with a 100× objective and switches between pairs of excitation (λ_ex_) and emission (λ_em_) wavelengths: Calbryte 520 (λ_ex_=488 nm, λ_em_=525 nm) and Mito-R-GECO1 (λ_ex_=561 nm, λ_em_=630 nm). Images were acquired at 1-s intervals for each indicator, corrected for background fluorescence, and analyzed using MetaMorph Microscopy Automation and Image Analysis software (Molecular Devices, San Jose, CA, USA). A region of interest was drawn around each cell successfully transfected with Mito-R-GECO1 and responses for both cytosolic and mitochondrial Ca^2+^ indicators were reported as F/F_max._ We confirmed that under the conditions used for recording [Ca^2+^]_c_ and [Ca^2+^]_m_, there was no bleed-through between the two channels (Fig. S1).

### Measurement of mitochondrial membrane potential

HeLa cells treated with NS siRNA or siRNA against KRAP were grown on poly-L-lysine-coated (0.01% w/v) 35-mm imaging dishes. Mitochondrial membrane potential was measured using a TMRE assay following the manufacturer's instructions. Briefly, cells were incubated with TMRE (100 nM, 15 min) and DAPI at 37°C in humidified air with 5% CO_2_. Where indicated, cells were incubated with FCCP (20 µM, 10 min) to dissipate the mitochondrial membrane potential before addition of TMRE. Cells were then washed twice with PBS, and fluorescence intensity was measured from single cells at 20°C in PBS using an EVOS M7000 cell imaging system (Thermo Fisher Scientific) equipped with a 60× oil-immersion objective (NA, 1.45). Cells were kept in the dark during all manipulations.

### Quantification of fluorescence images

Fluorescence images were corrected for background by subtraction of fluorescence collected from a region outside the cell using MetaMorph software. Capture and processing of widefield images and confocal PLA images was performed using MetaMorph. Confocal images of IP_3_R, KRAP and mitochondrial fluorescence were captured using Zen imaging software (Carl Zeiss Microscopy, Germany).

Colocalization analysis within widefield images (Mito-R-GECO1 and TOM20–GFP) used the JACoP plugin (Bolte and Cordelieres, 2006) in Fiji/ImageJ (Schindelin et al., 2012) to calculate the Manders' coefficients that report the fraction of Mito-R-GECO1 colocalized with TOM20–GFP. For colocalization analyses of confocal images, images were first deconvolved using SVI HuygensPro software (Scientific Volume Imaging B.V., VB Hilversum, the Netherlands) and then background corrected using MetaMorph. Colocalization used an object-based program within ImageJ (DiAna) (Gilles et al., 2017), wherein the centre-to-centre distances between each EGFP–IP_3_R punctum and the nearest KRAP punctum were assessed following a spot segmentation of each image. For analysis of three-colour colocalization (IP_3_Rs, KRAP and mitochondria), a mask of the regions containing mitochondria was generated, EGFP–IP_3_R puncta within these regions were identified and the centre-to-centre distances from these IP_3_R puncta to the nearest KRAP punctum were assessed using DiAna. We considered IP_3_R and KRAP puncta to be colocalized if their centre-to-centre distance was <233 nm (3 pixels), which is close to the theoretical lateral resolution of the confocal microscope.

PLA spots were quantified according to previously described methods (López-Cano et al., 2019). Briefly, following background subtraction, maximum *z*-projected images were converted into binary images. The number of PLA spots and cells in a field identified by DAPI staining were counted using the Analyze Particles function on ImageJ.

### Statistical analysis

Most results are presented as mean±s.d. or s.e.m. from *n* independent analyses. Statistical analyses used either two-tailed paired or unpaired Student's *t*-test (two variables) or one-way ANOVA with Tukey's multiple comparisons test (more than two variables); *P*<0.05 was considered significant (PRISM, version 9, GraphPad, USA). For colocalization analyses using DiAna, the distribution of KRAP puncta was shuffled 100 times within each cell and the distances from each KRAP punctum to the nearest IP_3_R punctum was reassessed. We considered the observed distances to be statistically significant if they fell outside the 95% confidence interval of the randomized distances.

## Supplementary Material

10.1242/joces.261728_sup1 Supplementary information
