# Supplementary material for: KRAP regulates mitochondrial Ca2+ uptake by licensing IP3 receptor activity and stabilizing ER–mitochondrial junctions
Source: J Cell Sci. 2024 Jun 27;137(12):jcs261728. doi: 10.1242/jcs.261728 (PMC11234384; doi:10.1242/jcs.261728)
Supplement: Supplementary information [file joces-137-261728-s1.pdf]

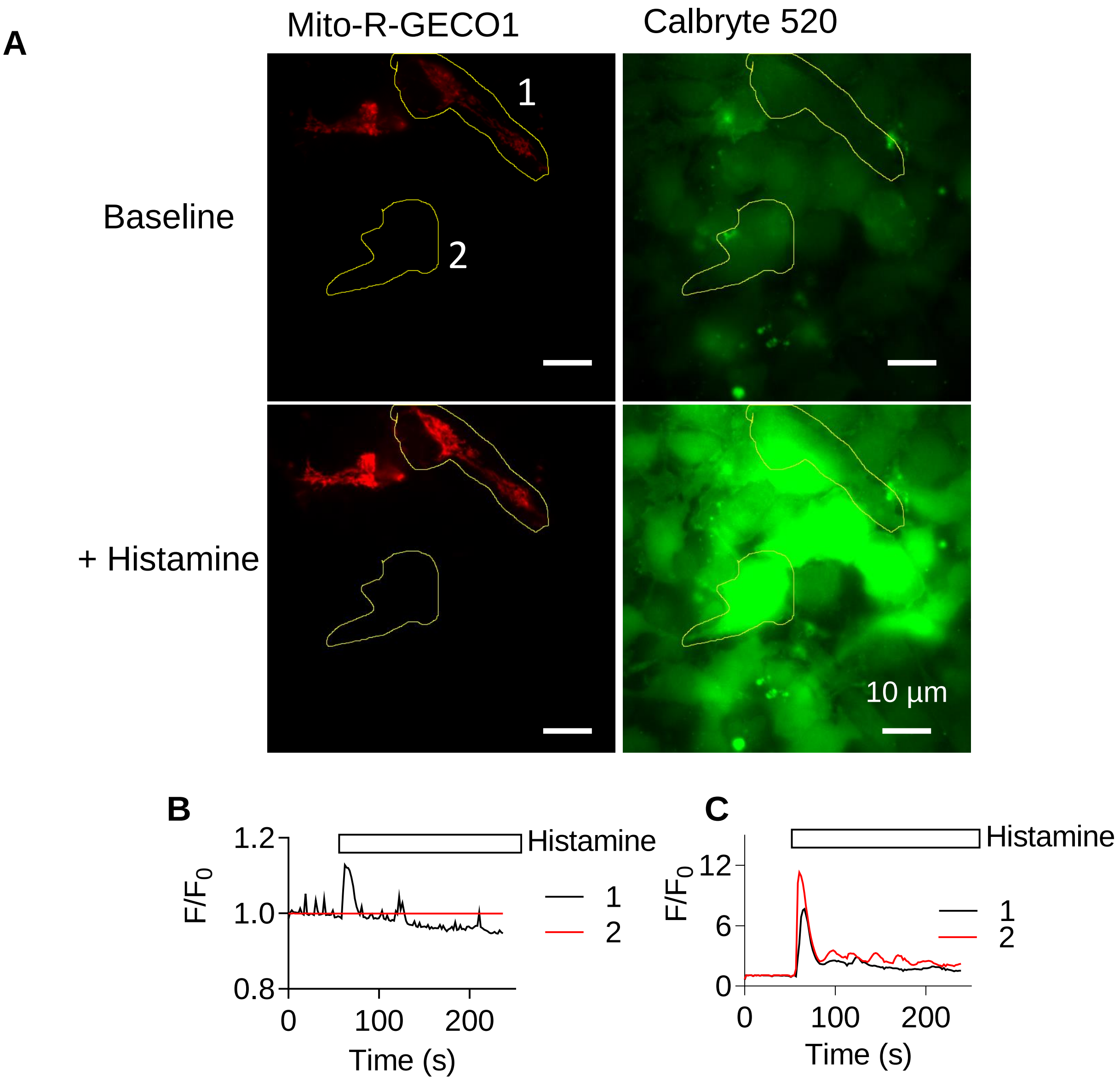

Fig. S1. There is no bleed-through between the Calbryte 520 and Mito-R-GECO1 channels. (A) Typical wide-field fluorescence images of HeLa cells expressing Mito-R-GECO1 (red) and loaded with Calbryte 520 (green), showing no crosstalk between channels before or after addition of histamine (100 µM). Cell boundaries (yellow lines) show a cell transfected with Mito-R-GECO1 and loaded with Calbryte 520 (1) and a cell not transfected with Mito-R-GECO1 but loaded with Calbryte 520 (2) in the same field of view. (B) Representative trace (F/F_0) from the red channel showing that only the cell transfected with Mito-R-GECO1 responds to histamine with a fluorescence change. (C) Representative trace from the green channel showing that both cells respond to histamine with fluorescence changes. The spectral properties of Calbryte 520 (excitation 493 nm, emission 515 nm) and Ca^{2+}-Mito-R-GECO1 (excitation 561 nm, emission 589 nm) and the filters used (525 nm, bandpass 50 nm, 630 nm, bandpass 75 nm) suggest crosstalk cannot occur from red to green.

Journal of Cell Science • Supplementary information

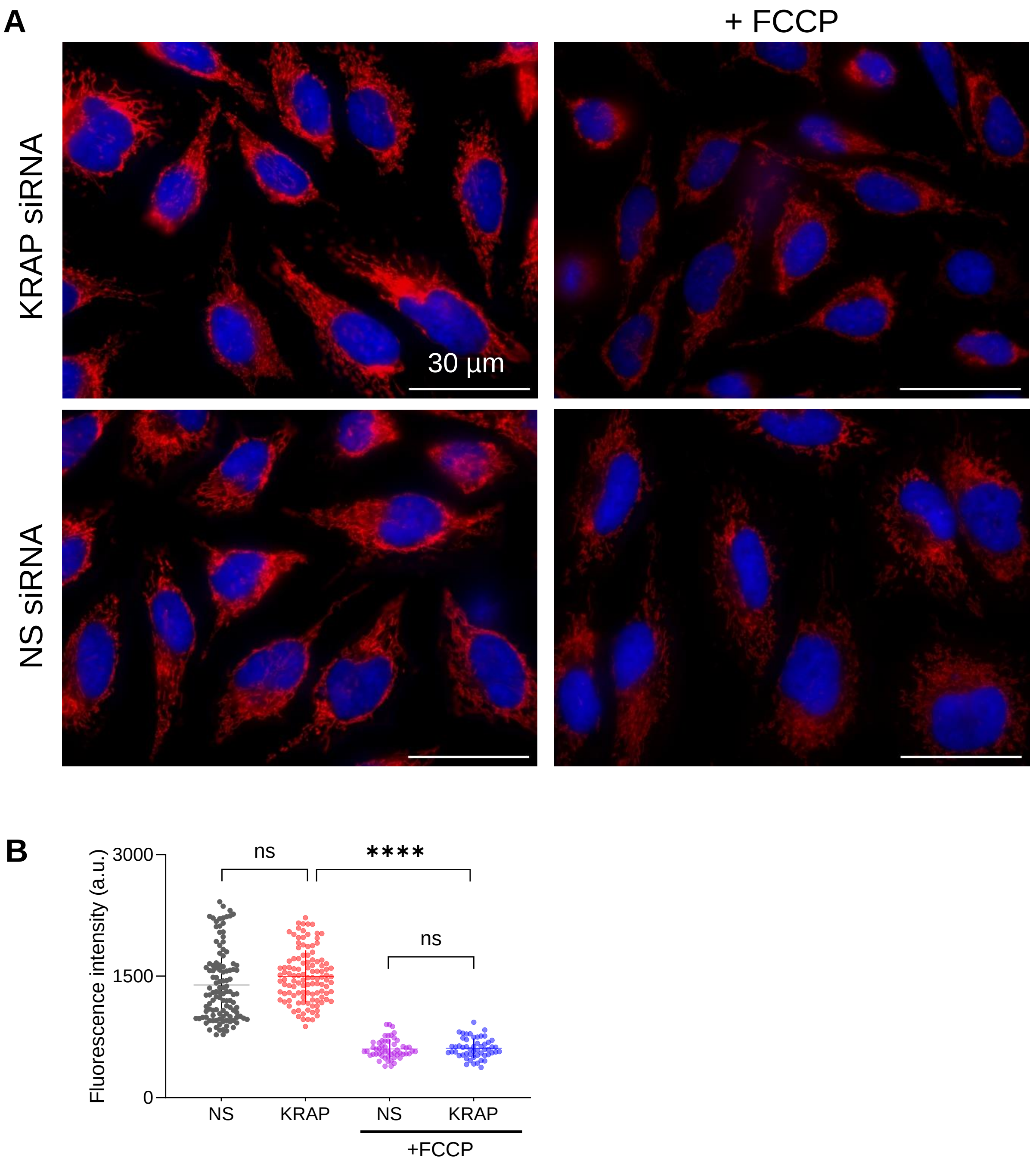

Fig. S2. Loss of KRAP does not affect mitochondrial membrane potential. (**A**) Wide-field fluorescence images of HeLa cells treated with siRNA against KRAP or non-silencing siRNA (NS) and stained after 72 hr with TMRE (100 nM, 15 min) to measure mitochondrial membrane potential (red). Cell nuclei are stained with DAPI (blue). FCCP (20 µM, 10 min) was used to collapse the mitochondrial membrane potential. (**B**) Summary results show the fluorescence intensity of TMRE staining (individual values, mean ± SD) from 114 (without FCCP) or 53 cells (with FCCP) from 3 independent experiments. ns $p > 0.05$; ****$p < 0.0001$, one-way ANOVA with Tukey correction.

Journal of Cell Science • Supplementary information

Table S1. Sequences of the siRNAs used.

Target	Sequence (5'→3')
KRAP	GCUAAAUGCAGUGAUAUGA
MCU (ON-TARGETplus SMARTpool)	
siRNA J-015519-17	GAUCAGGCAUUGUGGAAUA
siRNA J-015519-18	GUUUUGACCUAGAGAAAUA
siRNA J-015519-19	ACUGAGAGACCCAUUACAA
siRNA J-015519-20	GUAAUGACACGCCAGGAAU

Journal of Cell Science • Supplementary information
